# Transcatheter aortic valve replacement (TAVR) leads to an increase in the subendocardial viability ratio assessed by pulse wave analysis

**DOI:** 10.1371/journal.pone.0207537

**Published:** 2018-11-21

**Authors:** Claudia Müller, Georg Goliasch, Stefan Schachinger, Stefan Kastl, Thomas Neunteufl, Georg Delle-Karth, Johannes Kastner, Mariann Gyöngyösi, Irene Lang, Michael Gottsauner-Wolf, Noemi Pavo

**Affiliations:** 1 Department of Internal Medicine II, Division of Cardiology, Medical University of Vienna, Vienna, Austria; 2 Department of Internal Medicine I, Medical University Krems, Krems, Austria; 3 Department of Medicine IV, Hietzing, Vienna, Austria; University of Bern, University Hospital Bern, SWITZERLAND

## Abstract

**Background:**

Pulse wave analysis (PWA) is a useful tool for non-invasive assessment of central cardiac measures as subendocardial perfusion (Subendocardial Viability Ratio, SEVR) or contractility (dP/dt_max_). The immediate influence of transcatheter aortic valve replacement (TAVR) on these indices has not been investigated yet.

**Methods:**

We prospectively enrolled 40 patients presenting with severe aortic stenosis receiving TAVR. Central pressure curves were derived from radial and carotid sites using PWA up to 2 days before and 7 days after TAVR. Parameters were compared between peripheral measurement sites. Changes in SEVR, dP/dt_max_ and in indices of vascular stiffness were assessed. Additionally, association of these variables with clinical outcome was evaluated during a 12-month follow-up.

**Results:**

Central waveform parameters were comparable between measurement sites. SEVR, but not dP/dt_max_, augmentation Index (AIx) or augmentation pressure height (AGPH) correlated significantly with disease severity reflected by peak transvalvular velocity and mean transvalvular pressure gradient over the aortic valve (V_max_, ΔPm) [r = -0.372,*p* = 0.029 for V_max_ and r = -0.371,*p* = 0.021 for ΔPm]. V_max_ decreased from 4.5m/s (IQR:4.1–5.0) to 2.2m/s (IQR:1.9–2.7), (*p*<0.001). This resulted in a significant increase in SEVR [135.3%(IQR:115.5–150.8) vs. 140.3%(IQR:123.0–172.5),*p* = 0.039] and dP/dt_max_ [666mmHg(IQR:489–891) vs. 927mmHg(IQR:693–1092),*p*<0.001], and a reduction in AIx [154.8%(IQR:138.3–171.0) vs. 133.5%(IQR:128.3–151.8),*p*<0.001] and AGPH [34.1%(IQR:26.8–39.0) vs. 25.0%(IQR 21.8–33.7),*p* = 0.002], confirming the beneficial effects of replacing the stenotic valve. No association of these parameters could be revealed with outcome.

**Conclusions:**

PWA is suitable for assessing coronary microcirculation and contractility mirrored by SEVR and _max_dP/dt in the setting of aortic stenosis. PWA parameters attributed to vascular properties should be interpreted with caution.

## Introduction

Aortic stenosis is the most common valvular heart disease in elderly patients, with a prevalence of 2% to 7% above the age of 65, and is associated with a high mortality in untreated symptomatic patients.[1] Aortic stenosis is not only a valvular but also a left ventricular (LV) disease. The LV response to aortic stenosis is complex and partly influenced by intrinsic patient factors such as sex or comorbidities.[2] In general, pressure overload results in an increased wall stress, remodeling and ultimately myocardial hypertrophy conceivably in order to maintain systolic function, but represents rather a maladaptive mechanism.[3] Hypertrophy is associated with fibrosis and progressive fibrosis may lead to LV dilatation. Fibrosis is driven by several factors including an activated renin-angiotensin-aldosterone system or subendocardial ischemia.[4, 5] Patients suffering from aortic stenosis show a reduced coronary flow reserve even with angiographically normal coronary arteries,[5] most likely based on a reduced transmural perfusion gradient as a result of high LV end diastolic pressure. In the last decade, transcatheter aortic valve replacement (TAVR) became a generally accepted therapeutic alternative to open-heart surgery in intermediate to high risk patients with severe aortic stenosis.[6, 7] The 2-year follow-up (FUP) results of the PARTNER trial confirmed the sustained benefit of TAVR for high-risk patients, showing maintenance of the improvement in hemodynamics and reduction of clinical symptoms with a similar mortality when compared to surgery.[7] Regarding intermediate risk patients, the PARTNER 2 and the SURTAVI trial showed that TAVR was noninferior to surgical aortic valve replacement with respect to the primary composite endpoint of death or disabling stoke at 24 months using balloon-expandable and self-expanding prostheses.[8, 9]

Pulse wave analysis (PWA) is a simple technique offering the possibility of obtaining hemodynamic parameters non-invasively.[10] The recording of the pulse wave at the carotid or radial sites enables the construction of the wave contour of the ascending aorta by application of the transfer function. Analysis of this central curve yields basic parameters characteristic for the shape of the pulse wave, i.e. indication of pressures or time intervals, as well as parameters related to cardiac function, i.e. subendocardial perfusion (subendocardial viability ratio (SEVR) or contractility (dP/dt_max_), or vascular stiffness, i.e. augmentation index (AIx) and augmentation pressure height (AGPH). The SEVR is calculated based on systolic and diastolic pressures and their time integrals.[10] It represents the ratio between myocardial blood requirement and supply and reflects the degree of endomyocardial ischemia.[10] In hypertension, beneficial therapeutic effects of angiotensin-converting-enzyme inhibitors are accompanied by an increase in SEVR.[10] Echocardiography is the key diagnostic tool for the diagnosis of severe aortic stenosis and associated cardiac pathologies.[1] However, although multiple parameters can be obtained to estimate the severity of stenosis as the peak transvalvular velocity (V_max_), mean transvalvular pressure gradient (ΔPm) and the aortic valve area (AVA), echocardiographic measures are imperfect surrogates for wall stress and hemodynamic effects of the obstruction can not be characterized completely. In this setting PWA may provide additional hemodynamic indices thereby further characterizing severe aortic stenosis.

Although the hemodynamic effects of surgical aortic valve replacement have been investigated intensively there are only several reports on the effects of TAVR.[11] Especially, the diagnostic information measured by non-invasive PWA has not been reported in this setting. The aim of this study was to characterize periprocedural hemodynamic changes by using PWA. We hypothesized that TAVR would lead to immediate amelioration of PWA hemodynamic parameters.

## Methods

### Study population

We prospectively enrolled consecutive patients with aortic stenosis between July 2012 and August 2016 at the General Hospital of Vienna, a university-affiliated tertiary care center. Eligible patients were older than 18 years, suffered from severe symptomatic aortic stenosis and were considered for transfemoral TAVR by a heart team consisting of cardiologists and cardiac surgeons in line with the treatment guidelines. (11) Current medication as well as cardiac risk factors were recorded in all patients. Venous blood samples were obtained one day before the procedure and samples were analyzed according to our local laboratory standard procedures. Written, informed consent was obtained from all study participants. The study protocol complies with the Declaration of Helsinki and was approved by the local ethics committee of the Medical University of Vienna (EK 1834/2012).

### Echocardiography

Standard echocardiograms at baseline and after TAVR were performed using commercially available equipment (Vivid 7 and Vivid 9, GE Healthcare, Chicago, IL; Acuson Sequoia, Siemens, Berlin, Germany). Cardiac morphology was assessed using diameters and areas as well as volumetric measurements in standard 4‐ and 2‐chamber views.[12] Semiquantitative assessment of left ventricular function was performed by experienced readers using multiple acoustic windows and categorized into normal (≥55%), mildly reduced (45–54%), moderately reduced (30–44%), and severely reduced (<30%). Valve stenosis and regurgitation were quantified using an integrated approach and graded as none, mild, mild to moderate, moderate, moderate to severe, and severe according to current guidelines.[13, 14] Systolic pulmonary artery pressures were calculated by adding the peak tricuspid regurgitation systolic gradient to the estimated central venous pressure.

### Pulse wave analysis

Arterial PWA was performed using applanation tonometry directly before and up to seven days after TAVR. Central pressure curves were derived and analyzed after assessment of peripheral radial and carotid pressure curves using the SphygmoCor CvMS system (Atcor Medicals, Australia). For each patient multiple radial and carotid measurements were conducted by an experienced operator applying gentle pressure. Data of the averaged peripheral waveform and corresponding central waveform were collected directly in a portable microcomputer. The results of the best three measurements with an operator’s index above 80 were averaged. Peripheral and central waveforms were then analyzed using the system software to determine waveform characteristics. Besides time to first peak (T1), time to second peak (T2), pressure at T1 (P1) and pressure at T2 (P2) for each curve, the following parameters from the calculated central curves were obtained: subendocardial viability ratio (SEVR), maximal rate of rise of left ventricular pressure (dP/dt_max_), augmentation index (AIx) and augmentation pressure height (AGPH) as well as diastolic duration (DD), ejection duration per period (EDP).

### Statistical analysis

Continuous data were presented as median and IQR and categorical data as counts and percentages. PWA parameters before TAVR were correlated to echocardiographic parameters by calculating the Spearman´s correlation coefficient. To compare metrics before and after the TAVR procedure the Wilcoxon-test was used. Correlations between radial and carotid site measurements were analyzed by calculating the Pearson´s coefficient. For all tests two-sided p-values lower 0.05 were considered to indicate statistical significance.

## Results

### Baseline characteristics

A total of 50 consecutive patients were enrolled into this non-randomized single-center study. Ten patients either denied FUP or suffered from procedure related complications and were therefore excluded from the analysis. The detailed baseline characteristics are presented in *S1 Table*. The median age of patients was 83 years (IQR 80–87), and 18 (45%) patients were male. 12 (30%) patients had a moderately or severely reduced left ventricular function. The median STS-Score was 4.33% (IQR 2.90–4.56). Severe aortic stenosis was characterized by a median V_max_ of 4.5 cm/sec (IQR 4.1–5.0) and a median ΔPm of 47 mmHg (IQR 40–64). Edwards Lifesciences valves (Edwards Sapien XT or Edwards Sapien 3) were implanted in 36 cases, in 4 cases Medtronic Corevalve Evolut R were used.

### Echocardiographic parameters

*S2 Table* shows the echocardiographic parameters before and after the procedure. Due to TAVR the V_max_ [4.5m/s (IQR 4.1–5.0) vs. 2.2m/s (IQR 1.9–2.7), *p*<0.001] as well as ΔPm [47mmHg (IQR 40–64) vs. 9mmHg (IQR 8–14), *p*<0.001] were significantly reduced, as expected.

### Pulse wave analysis

**Fig 1:**
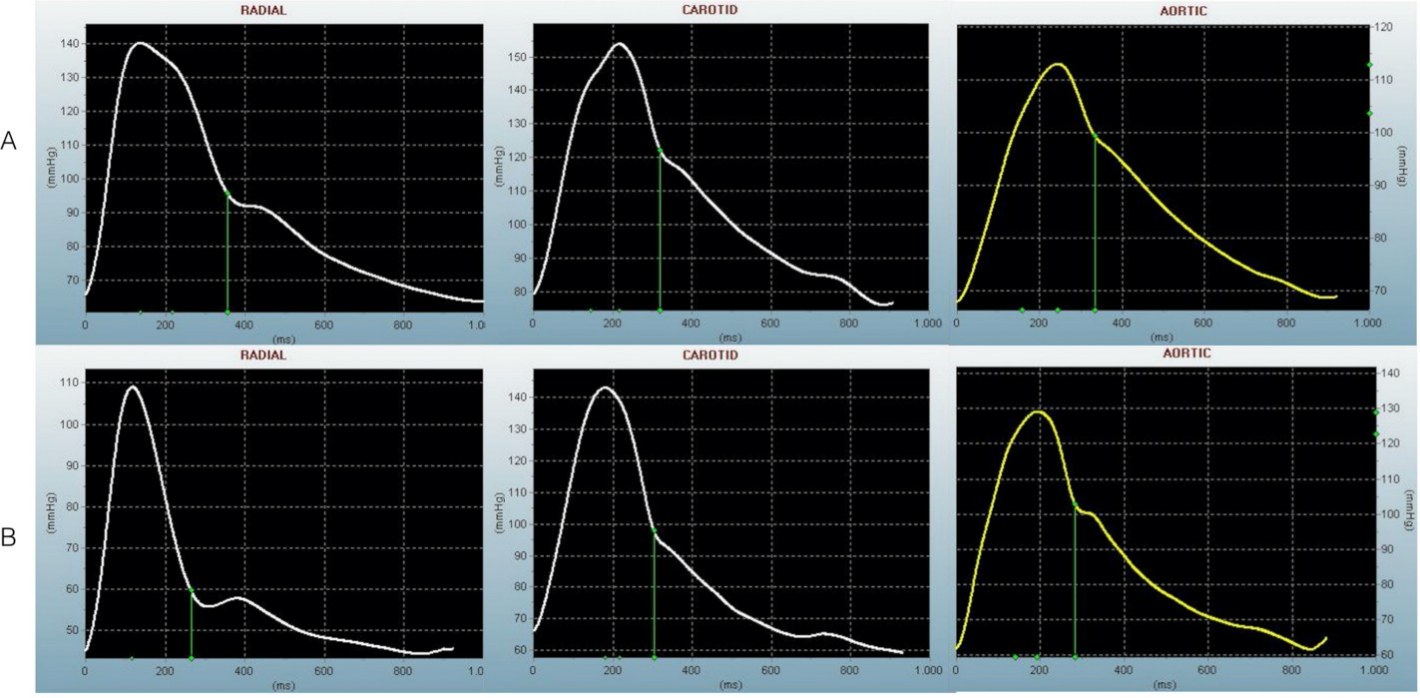
Characteristic pulse wave forms before and after TAVR. The results of pulse wave analysis recorded by applanation tonometry for a representative patient with severe aortic stenosis before (A) and after (B) TAVR are displayed. Radial and carotid measurements sites as well as calculated central curves are shown.

Characteristic peripheral and constructed central pressure wave curves for both sites are displayed in *Fig 1*, results are shown in *Table 1* and *S3 Table*. Both the peripheral and central curves are characterized by shorter times to maximal pressures (T1 and T2) alongside otherwise unchanged maximal pressures (P1 and P2) after TAVR. Similarly, ejection duration (ED) decreased significantly due to the procedure. Heart rate was similar for baseline and FUP measurements 70bpm (IQR 65–78) vs 70bpm (IQR 60–79), p = 0.252].

**Table 1:** Results of the pressure wave analysis (PWA) for patients with severe aortic stenosis undergoing transfemoral TAVR procedure. Characteristic parameters of the calculated central pressure waves from measurements of both radial and carotid sites are displayed. Variables are given as median and IQR. Comparison of the parameters before and after TAVR was performed using the Wilcoxon-test. Correlation between all measurements for radial and carotid sites were assessed by calculating the Pearson´s correlations coefficient. P-values <0.05 indicate statistical significance.

	Radial site			Carotid site				
	Before TAVR	After TAVR	*p*-value	Before TAVR	After TAVR	*p*-value	r	*p*-value
C_T1, ms (IQR)	110.7 (102.8–127.0)	103.3 (94.8–110.7)	**0.002**	104.6 (94.0–145.7)	86.6 (80.8–94.3)	**<0.001**	0.409	**<0.001**
C_T2, ms (IQR)	221.9 (203.0–237.5)	199.0 (187.5–214.3)	**<0.001**	232.4 (213.7–254.0)	197.3 (192.1–216.9)	**<0.001**	0.710	**<0.001**
C_P1, mmHg (IQR)	106 (95–117)	104 (91–118)	0.933	111 (102–122)	113 (102–129)	0.116	0.906	**<0.001**
C_P2, mmHg (IQR)	120 (108–132)	113 (102–133)	0.328	130 (120–148)	130 (120–149)	0.673	0.964	**<0.001**
C_AIx, % (IQR)	154.8 (138.3–171.0)	133.5 (128.3–151.8)	**<0.001**	161.5 (144.4–176.5)	139.5 (127.1–158.0)	**0.002**	0.634	**<0.001**
C_AP, mmHg (IQR)	15.1 (11.0–20.2)	11.5 (8.0–18.0)	**0.009**	20.5 (17.0–23.2)	16.3 (11.2–23.2)	**0.036**	0.805	**<0.001**
C_AP75, mmHg (IQR)	15.6 (10.7–18.7)	11.6 (7.3–16.8)	**0.002**	17.7 (13.5–23.5)	15.9 (9.0–21.0)	0.116	0.715	**<0.001**
C_AGPH, % (IQR)	34.1 (26.8–39.0)	25.0 (21.8–33.7)	**0.002**	35.3 (29.2–40.0)	28.0 (20.7–36.5)	**0.012**	0.645	**<0.001**
C_AGPH75, % (IQR)	33.7 (28.7–39.0)	27.0 (19.7–32.3)	**<0.001**	31.3 (24.4–39.3)	28.3 (17.5–35.3)	**0.036**	0.577	**<0.001**
C_TTI, mmHg s min^-1^	2342 (2062–2765)	2036 (1790–2659)	**0.022**	2500 (2186–2993)	2359 (1983–2835)	0.108	0.811	**<0.001**
C_DTI, mmHg s min^-1^	3160 (2888–3443)	3233 (2836–3488)	0.769	3175 (2944–3634)	3431 (2986–3641)	0.055	0.781	**<0.001**
C_SEVR, % (IQR)	135.3 (115.5–150.8)	140.3 (123.0–172.5)	**0.039**	131.6 (111.0–145.0)	137.5 (118.5–169.3)	**0.007**	0.686	**<0.001**
C_ESP, mmHg (IQR)	108 (97–118)	104 (94–117)	0.185	115 (104–121)	107 (100–116)	0.624	0.942	**<0.001**
C_DD, ms (IQR)	537.0 (440.5–606.0)	555.0 (487.7–634.0)	0.802	563.3 (460.0–641.3)	566.5 (460.8–683.3)	0.405	0.741	**<0.001**
C_adjED, ms (IQR)	320.7 (303.8–340.8)	291.2 (272.3–314.7)	**<0.001**	327.3 (301.8–345.7)	290.9 (273.4–318.2)	**<0.001**	0.540	**<0.001**
P_dP/dtmax*, mmHg/ms (IQR)	666 (489–891)	927 (693–1092)	**<0.001**	570 (435–692)	919 (703–1146)	**<0.001**	0.841	**<0.001**

AGPH—Augemntation/Pulse Height; AI–augmentation index; DD–diastolic duration,; ED period–ejection duration/period; P1 –pressure at T1; P2 –pressure at T2; SEVR–subendocardial viability ratio; fT1 –time to first peak; T2 –time to second peak; onts in bold indicate statistical significance (*p*<0.05).

* directly derived from the peripheral curves

At baseline SEVR but not dP/dt_max_, AIx or AGPH correlated significantly with echocardiographic [r = -0.37, *p* = 0.029 for V_max_ and r = -0.37, *p* = 0.027 for P_mean_] reflecting disease severity.

**Fig 2:**
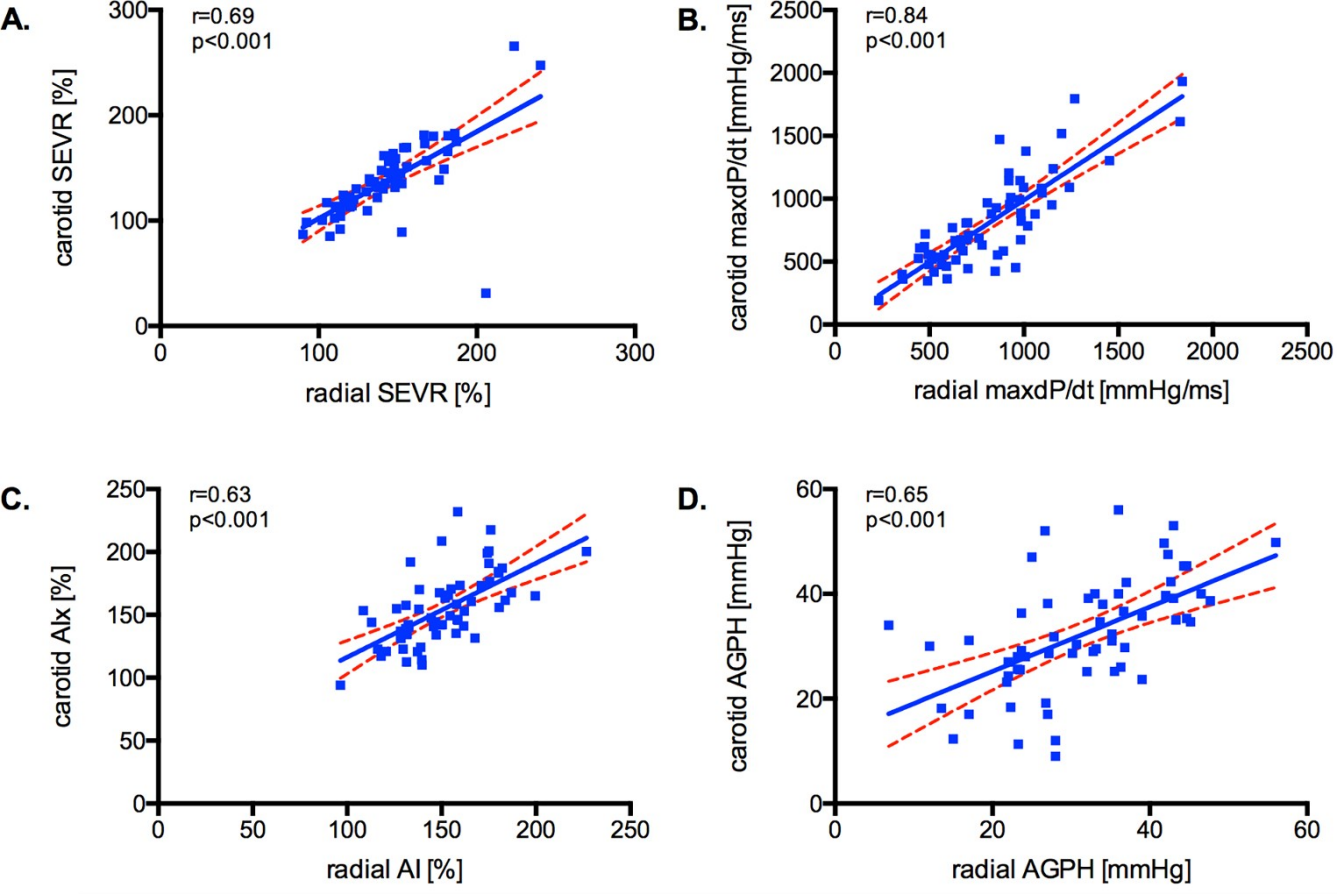
Correlation for PWA measures between peripheral measurement sites. The results for the calculated parameters SEVR (A), dP/dt_max_ (B), AIx (C) and AGPH (D) are displayed. Linear regression was performed between the two peripheral measurement sites, 95% confidence intervals and the correlation coefficients are indicated.

The calculated central parameters obtained from radial and carotid site measurements yielded similar results with highly significant excellent correlation coefficients [r = 0.41 for T1, r = 0.71 for T2; r = 0.91 for P1; r = 0.96 for P2; r = 0.84 for dP/dt_max_; r = 0.69 for SEVR; r = 0.63 for AIx and r = 0.65 for AGPH; *p*<0.001 for all]. The good comparability between the two sites for the parameters of interest is graphically displayed in *Fig 2*.

**Fig 3:**
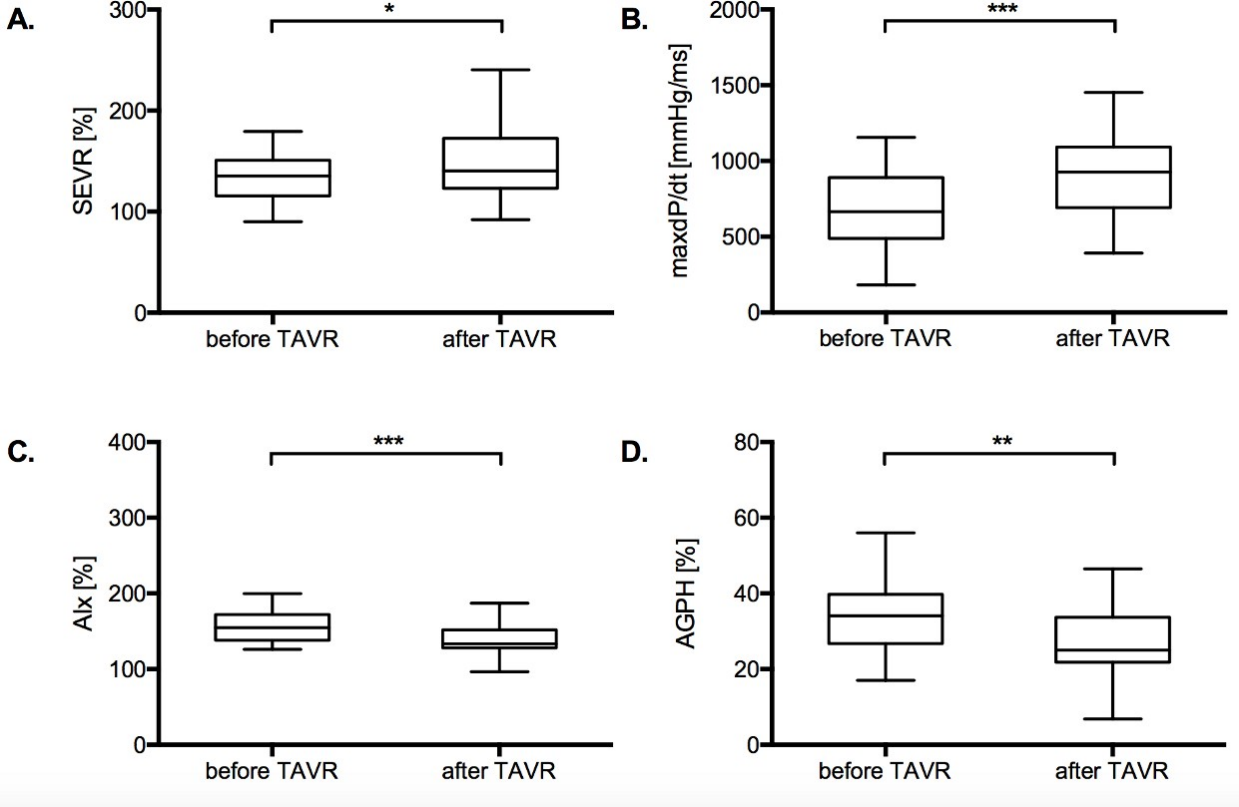
Cardiac performance and vascular stiffness indices before and after TAVR. The results for the calculated parameters SEVR (A), dP/dt_max_ (B), AIx (C) and AGPH (D) are displayed as Tukey plots. Medians were compared by the Wilcoxon test.

As a result of the procedure the SEVR improved significantly [135.3% (IQR 115.5–150.8) vs. 140.3% (IQR 123.0–172.5), *p* = 0.039 for the radial site and 131.6% (IQR 111.0–145.0) vs. 137.5% (IQR 118.5–169.3), *p* = 0.007 for the carotid site]. Similarly, the dP/dt_max_ [666mmHg/ms (IQR 489–891) vs. 927mmHg/ms (IQR 693–1092), p<0.001 for the radial site and 570mmHg/ms (IQR 435–692) vs. 919mmHg/ms (IQR 703–1146), *p*<0.001 for the carotid site] as well as the AIx [154.8% (IQR 138.3–171.0) vs. 133.5% (IQR 128.3–151.8); *p*<0.001 for the radial site and 161.5% (IQR 144.4–176.5) vs. 139.5% (127.1–158.0); *p* = 0.002 for the carotid site] and the AGPH [34.1% (IQR 26.8–39.0) vs. 25.0% (IQR 21.8–33.7); *p* = 0.002 for the radial site and 35.3% (IQR 29.2–40.0) vs. 28.0% (20.7–36.5); *p* = 0.012 for the carotid site] improved significantly. The results are depicted in *Fig 3*.

Comparison of PWA parameters at baseline and at FUP for subgroups according to LVEF, type of aortic stenosis, i.e. normal flow true severe AS vs low-flow low-gradient AS or paradoxical low-flow low-gradient AS, severity of mitral regurgitation or valve type implanted yielded similar results.

## Discussion

This is the first study to assess hemodynamic changes by PWA using non-invasive applanation tonometry periprocedural in the setting of TAVR. Our results show that PWA yields comparable results for central curves independently from the measurement site, i.e. carotid or radial. Measurements of endomyocardial perfusion and left ventricular contractility (reflected by SEVR and dP/dt_max_) improve significantly following TAVR procedure. Additionally, the commonly used parameters estimating vascular stiffness as AIx or AGPH change significantly. No association for baseline or follow-up SEVR, dP/dt_max_, AIx or AGPH could be proven with all-cause mortality. AIx and AGPH as measures for vascular stiffness should have adjusted cut-offs in the setting of significant aortic stenosis.

Concerning pulse and pressure measurements, most studies and a recent meta-analysis suggest that central pressures are better predictors of cardiovascular risk than peripheral arterial pressures.[15] Applanation tonometry is a technique enabling the calculation of a central waveform after recording an easily accessible peripheral waveform. Thereby the central aortic pressure curve becomes available in a non-invasive fashion obviating the need for cardiac catheterization for this purpose. In this study, central aortic pressures were derived by the application of a transfer function, that has been proven to generate accurate estimates of the central arterial pressure waveform and its properties.[16] Parameters derived from the radial and carotid site showed an excellent correlation. This indicates that radial measurements alone may be used for the estimation of central hemodynamics without the need of performing the practically more challenging carotid measurements.

### PWA-derived cardiac measures

Our data show, that TAVR procedure ultimately leads to an immediate improvement of the central curve characteristics reflected by higher contractility and perfusion parameters. The increase in dP/dt_max_ seems reasonable, since the left ventricular myocardial tissue is able to realize a better cardiac performance after the relieve of the obstruction. PWA allows the approximation of SEVR, also known as Buckberg index, a parameter reflecting the relation of myocardial oxygen supply and demand.[17, 18] SEVR was originally defined as the ratio of diastolic and systolic pressure-time integrals from measurements of the aorta and the LV, i.e. area between the aortic and LV pressures in diastole divided by the area under the LV pressure curve in systole, thereby relating subendocardial blood supply to myocardial contraction.[18, 19] In normal coronary arteries the critical value of SEVR below which subendocardial ischaemia occurs was reported as 50%.[19] Here, the tonometric SEVR was calculated by the manufacturer as diastolic aortic area / systolic aortic area. Our data reveal a significant increase in SEVR after TAVR suggesting amelioration of myocardial blood supply after replacement of the stenotic valve. Additionally, the SEVR correlated significantly with the echocardiographic parameters V_max_ and the ΔPm, both strong predictors for clinical outcome for symptomatic as well as asymptomatic patients suffering from aortic stenosis.[20, 21] Up to date there is only one report of pulse waves in the setting of percutaneous aortic valve replacement.[11] This study investigated invasively assessed coronary pulse waves calculating the coronary suction wave and found an impaired physiologic coronary reserve with severe aortic stenosis and normalization of the pulse wave pattern after TAVR.[11] Impaired coronary suction wave was subsequently suggested to be one pathophysiologic correlate to angina symptoms.[11] Another study investigating hypertensive subjects without significant coronary lesions showed that SEVR, determined by the same system as used in our study, was the only independent predictor among PWA parameters for coronary flow reserve.[18] These findings are in line with our pathophysiologic understanding and our data highlight the utility of SEVR as an easily accessible parameter of myocardial viability in the setting of aortic stenosis.

### PWA-derived indices of vascular stiffness

The central AIx, which is calculated as the ratio of augmentation pressure to central pulse pressure, was originally thought to be an index of peripheral wave reflection.[22] Due to degeneration or hyperplasia in the arterial wall arterial stiffness increases causing the reflected wave to arrive earlier in the central aorta thereby augmenting pressure in the late systole.[23] However recent works assume that AIx might rather reflect the reservoir function of the aorta.[15] Nevertheless central AIx is an independent predictor for cardiovascular events.[24, 25] One explanation is that AIx reflects arterial wall dysfunction which itself is associated with deleterious loading conditions of the left ventricle. An increased ventricular afterload would result in a decline in myocardial perfusion accompanied by elevated myocardial oxygen demand.[23] Increased systolic pressures promote atherosclerosis at the same time.[23] AIx has been shown to be dependent on heart rate with a strong inverse correlation in a study investigating the influence by using cardiac pacing as well as in hypertensive subjects. [26, 27] Interpretation of inter-individual differences in AIx or using it as an index of arterial stiffness should be done with caution under conditions of varying heart rates.[28]

The interest in central AIx as a prognostic marker lead to the derivation of normative values from large population studies including a total of 10550 subjects to allow for comparison.[29] The utility of central AIx for risk stratification and targeted therapeutic approaches will be investigated in future studies. According to this study the equation for central AIx for white British men and women in supine position is 148*Age^0.267^*H^−0.576^*HR^−0.215^ and 121*Age^0.319^*H^−0.594^*HR^−0.196^, respectively. Using this formula with the median values of baseline characteristics for our population results in an estimated central AIx of 152.89% (IQR 141.48–165.62). This value is closer to our measurements after TAVR than at baseline.

Although the study by Chirinos et al. investigated associations between central AIx and comorbidities or ethnicity data,[29] the influence of valvular disease, especially aortic stenosis, on central AIx and other PWA parameters is lacking. To our knowledge this is the first study assessing PWA in patients with severe aortic stenosis undergoing replacement of the stenotic valve offering the possibility to picture the effects of aortic stenosis on PWA parameters. The observation that central AIx and AGPH change significantly during the procedure challenge the interpretation of these parameters in the setting of aortic stenosis. In our current understanding both parameters should reflect mere vascular properties, however, our results show, that these parameters will be overestimated in the setting of significant aortic stenosis.

### Limitations

One potential limitation of our study is that our data represent the experience of a single tertiary care center. Therefore, a center-specific bias cannot be excluded, and all results and conclusions should be interpreted with caution. However, the major advantages of limiting data collection to a single center are the inclusion of a more homogenous patient population, adherence to a consistent clinical routine, as well as a consistent quality of imaging, PWA and TAVR procedures. Another limitation may be the different valve types used. Moreover, we have included all patients referred to TAVR depicting a real-life scenario, however the investigation of distinct subgroups, e.g. type of aortic stenosis or left ventricular systolic function, should be pursued in further studies with larger cohorts. Lastly, a higher number of patients could enable an additional evaluation of the impact on clinical outcome and mortality.

## Conclusion

In conclusion, our study provides evidence that PWA is a useful tool for assessing coronary microcirculation by assessing SEVR. PWA parameters attributed to vascular properties should be interpreted with caution in the setting of significant aortic stenosis. Future studies with bigger cohorts might investigate the relevance of PWA parameters in aortic stenosis and potentially establish SEVR as a parameter for patient selection or prediction of prognosis.

## Supporting information

S1 Table Baseline characteristics of patients receiving transfemoral TAVR (n = 40). (DOCX)

S2 Table Echocardiographic parameters before and after the TAVR procedure. (DOCX)

S3 Table Results of the pressure wave analysis (PWA) for patients with severe aortic stenosis undergoing a transcatheter aortic valve replacement (TAVR) procedure. (DOCX)
