# Supplementary material for: Transcatheter aortic valve replacement (TAVR) leads to an increase in the subendocardial viability ratio assessed by pulse wave analysis
Source: PLoS One. 2018 Nov 21;13(11):e0207537. doi: 10.1371/journal.pone.0207537 (PMC6248990; doi:10.1371/journal.pone.0207537)
Supplement: S1 Table — (DOCX) [file pone.0207537.s001.docx]

**Supporting Information**

**S1 Table.** Baseline characteristics of patients receiving transfemoral TAVR (n=40). Continuous variables are given as medians and inter-quartile ranges (IQR). Counts are given as numbers and percentages.

|  | **TAVR patients (n=40)** |
| --- | --- |
| Age, years (IQR) | 83 (80-87) |
| Male gender, n (%) | 18 (45%) |
| BMI kg/m^2^, (IQR) | 25.6 (21.9–29.2) |
| Valve type |  |
| Edwards sapien XT, n (%) | 15 (37.5%) |
| Edwards sapien 3, n (%) | 21 (52.5%) |
| Evolut R, n (%) | 4 (10%) |
| Comorbidities |  |
| Significant CAD, n (%) | 25 (62.5%) |
| Stroke / TIA, n (%) | 0 (0%) |
| Significant PAD, n (%) | 6 (15%) |
| Diabetes mellitus, n (%) | 8 (20%) |
| Arterial Hypertension, n (%) | 30 (75%) |
| COPD, n (%) | 3 (7.5%) |
| Currently smoking, n (%) | 3 (7.5%) |
| Sinusrhythm, n (%) | 27 (67.5%) |
| PM or ICD (+/- CRT), n (%) | 3 (7.5%) |
| Medication |  |
| Beta-Blocker, n (%) | 21 (52.5%) |
| ACE-I, n (%) | 15 (37.5%) |
| ARB, n (%) | 9 (22.5%) |
| MRA, n (%) | 14 (35%) |
| Digitalis, n (%) | 1 (2.5%) |
| ASS, n (%) | 25 (62.5%) |
| Clopidogrel, n (%) | 22 (55%) |
| Oral anticoagulation VKA, n (%) | 10 (25%) |
| Oral anticoagulation NOAC, n (%) | 3 (7.5%) |
| Echocardiographic parameters |  |
| Vmax, m/s (IQR) | 4.5 (4.1-4.95) |
| ∆Pm, mmHg (IQR) | 47 (40-64) |
| AVA, cm^2^ (IQR) | 0,6 (0.5-0.7) |
| LV diameter, mm (IQR) | 44 (41-50.25) |
| LVF normal, n (%) | 25 (62.5%) |
| LVF mildly reduced, n (%) | 2 (5%) |
| LVF moderately or severely reduced, n (%) | 12 (30%) |
| MR grade moderate or severe, n (%) | 19 (47.5%) |
| IVS, mm (IQR) | 14.5 (13-16) |
| TR grade moderate or severe, n (%) | 19 (47.5%) |
| sPAP, mmHg (IQR) | 56 (41.5-65.5) |
| Labor parameters |  |
| Serum creatinine, mg/dl (IQR) | 1.02 (0.85-1.32) |
| GFR, mL/min/1.73 m^2^ (IQR) | 44.31 (35.89-60.84) |
| NT-proBNP, pg/ml (IQR) | 1831 (830-4655) |
| CRP, mg/l (IQR) | 0.44 (0.15–1.15) |
| Normotest, % (IQR) | 80 (64.0-93.0) |
| INR, (IQR) | 1.2 (1.0-1.45) |
| aPTT, sec (IQR) | 36.2 (32.98-41.5) |
| Hb, mg/dl (IQR) | 11.15 (9.43–12.8) |
| Leukocytes, 1000/µl (IQR) | 6.18 (5.56-7.45) |
| Cholinesterase U/ml (IQR) | 6.02 (4.7-7.0) |
| GOT, U/l (IQR) | 22 (19.0-27.8) |
| GPT, U/l (IQR) | 16.5 (12.0-27.75) |
| Gamma-GT, U/l (IQR) | 22.5 (17.0-69.25) |

ACE-I – angiotensin converting enzyme inhibitor; aPTT - activated partial thromboplastin time; ARB – angiotensin receptor blocker; ASS – acetyl salicylic acid; AVA - aortic valve area; BMI – body mass index, CAD – coronary artery disease; COPD – chronic obstructive pulmonary disease; CRP - C-reactive protein; Gamma-GT - gamma glytamyl transpeptidase; GOT - glutamyl oxaloacetic transaminase; GPT - glutamyl pyruvic transaminase; GFR – glomerular filtration rate; Hb - hemoglobin; ICD – inter cardiac defibrillator; ; INR - international normalized ratio; IVS - interventricular septum; IQR – interquartile range; LV - left ventricular; LVF - left ventricular function; MR - mitral regurgitation; MRA - mineralocorticoid receptor antagonist; NOAC – non-vitamin K antagonist oral anticoagulant; NT-proBNP – N-terminal pro B-type natriuretic peptide; PAD – peripheral artery disease; ∆Pm - mean aortic transvalvular pressure gradient; PM – pacemaker; sPAP - systolic pulmonary artery pressure; TIA – transitory ischemic attack; TR - tricuspid regurgitation; VKA – vitamin K antagonist; Vmax - peak aortic transvalvular velocity
