# Supplementary material for: Transcatheter aortic valve replacement (TAVR) leads to an increase in the subendocardial viability ratio assessed by pulse wave analysis
Source: PLoS One. 2018 Nov 21;13(11):e0207537. doi: 10.1371/journal.pone.0207537 (PMC6248990; doi:10.1371/journal.pone.0207537)
Supplement: S2 Table — (DOCX) [file pone.0207537.s002.docx]

**Supporting Information**

**S2 Table.** Echocardiographic parameters before and after the TAVR procedure. Variables are displayed as median and inter quartile range (IQR).

|  | **Before TAVR** | **After TAVR** | ***p*-value** |
| --- | --- | --- | --- |
| V_max_, m/s (IQR) | 4.5 (4.1-5.0) | 2.2 (1.9-2.7) | **<0.001** |
| ∆Pm, mmHg (IQR) | 47 (40-64) | 9 (8-14) | **<0.001** |
| LV diameter, mm (IQR) | 44 (41-50) | 44 (40-47) | 0.242 |
| IVS, mm (IQR) | 15 (13-16) | 14 (13-16) | 0.339 |
| sPAP, mmHg (IQR) | 56 (42-66) | 53 (37-67) | 0.093 |

AV_max_ - peak jet velocity; IVS - interventricular septum; LV - left ventricular; P_mean_ - mean pressure gradient; sPAP - systolic pulmonary artery pressure
