# Supplementary material for: Transcatheter aortic valve replacement (TAVR) leads to an increase in the subendocardial viability ratio assessed by pulse wave analysis
Source: PLoS One. 2018 Nov 21;13(11):e0207537. doi: 10.1371/journal.pone.0207537 (PMC6248990; doi:10.1371/journal.pone.0207537)
Supplement: S3 Table — (DOCX) [file pone.0207537.s003.docx]

**Supporting Information**

**S3 Table.** Results of the pressure wave analysis (PWA) for patients with severe aortic stenosis undergoing a transcatheter aortic valve replacement (TAVR) procedure. Characteristic parameters of the peripheral pressure waves from measurements of both radial and carotid sites are displayed. Variables are given as median and IQR. Comparison of the parameters before and after TAVR was performed using the Wilcoxon-test.

|  | **Radial site** | | | **Carotid site** | | |
| --- | --- | --- | --- | --- | --- | --- |
|  | Before TAVR | After TAVR | *p*-value | Before TAVR | After TAVR | *p*-value |
| P_T1, ms (IQR) | 133 (121-155) | 119 (109-127) | **<0.001** | 125 (110-141) | 97 (84-111) | **<0.001** |
| P_T2, ms (IQR) | 197 (188-209) | 183 (162-200) | **0.004** | 226 (194-249) | 185 (171-200) | **<0.001** |
| P_P1, mmHg (IQR) | 125 (116-140) | 130 (114-149) | 0.261 | 118 (112-133) | 122 (110-144) | **0.050** |
| P_P2, mmHg (IQR) | 128 (116-142) | 121 (110-137) | 0.872 | 132 (123-152) | 135 (123-151) | 0.702 |
| P_AIx, % (IQR) | 101.1 (92.8-107.5) | 92.0 (78.0-106.0) | **0.001** | 133.8 (115.5-144.1) | 119.0 (106.0-138.2) | **0.018** |
| P_dP/dtmax, mmHg/ms (IQR) | 666 (489-891) | 927 (693-1092) | **<0.001** | 570 (435-692) | 919 (703-1146) | **<0.001** |

AI - augmentation index; maxdP/dt - peripheral pulse maximum dP/dt; P1 – pressure at T1; P2 – pressure at T2; T1 – time to first peak; T2 – time to second peak
